# Global, regional, and national burden and trends of bladder cancer in individuals aged 55 years and older from 1990 to 2021: findings from the Global Burden of Disease Study 2021

**DOI:** 10.1097/JS9.0000000000003254

**Published:** 2025-08-21

**Authors:** Haodong Li, Shengtao Zeng, Chenglin Yang, Yue Yang, Zhi Cao, Zhengfei Hu, Yuansong Xiao, Xiaoming Zhang, Wei Wang

**Affiliations:** a Department of Urology, General Hospital of Southern Theater Command, Guangzhou, China; b The First School of Clinical Medicine, Southern Medical University, Guangzhou, China

**Keywords:** bladder cancer, disability-adjusted life years, frontier analysis, Global Burden of Disease, Joinpoint analysis, prediction analysis

## Abstract

**Background:**

Bladder cancer (BC), a prevalent genitourinary malignancy, poses a substantial threat to public health, particularly among middle-aged and elderly populations. Given the increasing life expectancy, understanding the BC burden in these demographic groups is imperative.

**Methods:**

Based on the GBD 2021 dataset encompassing 371 diseases and injuries with 88 risk factors globally, this study analyzed bladder cancer data from 204 countries and territories (1990–2021) for populations aged ≥55 years, with core metrics including incidence, deaths, and disability-adjusted life years (DALYs). We used Joinpoint regression analysis to assess temporal trends, calculating the Annual Percentage Change (APC) and Average Annual Percentage Change (AAPC). We conducted a decomposition analysis to break down the changes in mortality rates into contributions from aging, population growth, and epidemiological changes. We performed a frontier analysis using the Socio-demographic Index (SDI) to evaluate the relationship between bladder cancer burden in people aged 55 and above and socio-demographic development. Among them, we further combined the National Health and Nutrition Examination Survey (NHANES) database for risk analysis related to bladder cancer. Finally, we used the Bayesian age-period-cohort (BAPC) model to project bladder cancer trends from 2022 to 2036.

**Results:**

From 1990 to 2021, bladder cancer cases among adults ≥55 years increased by 113% (226 421 to 483 234), while crude incidence rates declined slightly. Mortality rates decreased from 16.83 to 14.11 per 100 000, and DALYs rates dropped from 337.59 to 257.76. High-income regions (North America: 76.85/100 000; Western Europe: 77.62/100 000) had the highest incidence but showed downward trends, whereas Central Europe experienced rising mortality. Males consistently bore a 3.8-fold higher incidence burden than females. Smoking (26.6%) and high fasting glucose (8.2%) were key risk factors. According to NHANES data, elevated fasting blood glucose levels and smoking are both associated with an increased risk of bladder cancer. Projections to 2036 suggest continued declines: incidence (−13.3%), mortality (−16.4%), and DALYs (−17.9%). Health inequality persisted but improved marginally.

**Conclusion:**

The current data and future prediction of this study show that the incidence rate, mortality and DALYs rate of bladder cancer in middle-aged and elderly people have declined. However, disparities in disease burden still exist across regions. Thus, tailored measures are needed to reduce inequalities in high-burden areas.

## Introduction

Bladder cancer ranks as the ninth most commonly diagnosed malignancy globally, with approximately 614 000 new cases and 220 000 deaths reported in 2022. Although the incidence rate among males has shown a relative decline compared to females in recent years, the disease burden of bladder cancer remains disproportionately higher in men worldwide^[1]^, reflecting persistent gender disparities in risk exposure and biological susceptibility. According to World Health Organization projections, the number of bladder cancer cases is expected to potentially double by 2040^[2]^. Major risk factors for bladder cancer include tobacco smoking, occupational exposures (e.g., aluminum/dye production), environmental hazards (ionizing radiation, arsenic), specific medications (cyclophosphamide, phenacetin), opioid use, and Schistosoma infection. These factors have been confirmed as significant contributors to bladder cancer development^[3,4]^. Diagnostic evaluations reveal that 25–30% of bladder cancer patients present with tumor invasion into deeper bladder wall layers (muscle-invasive bladder cancer, MIBC) or metastatic disease at initial diagnosis^[5]^. If left untreated, the majority of patients with MIBC succumb to the disease within 2 years of diagnosis^[6]^. This suggests that bladder cancer is one of the cancers that leads to a significant financial burden, placing a huge strain on both the individual patient and the healthcare system^[7]^.

Population aging significantly challenges bladder cancer care due to aggressive tumor biology, advanced-stage diagnosis, and limited treatment options in elderly patients^[8]^. Older individuals are less likely to receive radical cystectomy for MIBC compared to younger counterparts^[9]^. The Global Burden of Disease (GBD) report indicates that the relative risk of incidence and mortality for ages 15–19 is the lowest and then increases with age^[10,11]^. Some studies have shown that in patients with nasopharyngeal carcinoma or astrocytoma, individuals aged 55 years and older tend to have a relatively poor prognosis^[12,13]^. Therefore, in-depth temporal trend analysis, geographic variation studies, and risk factor assessments of bladder cancer in middle-aged and older populations are critically important for developing effective public health strategies and targeted interventions.

It is critical to emphasize that no prior studies have systematically investigated the epidemiology of bladder cancer among individuals aged ≥55 years using the GBD 2021 dataset, leaving a significant evidence gap in understanding age-specific burden patterns and intervention priorities for this high-risk demographic. We conducted an in-depth analysis of bladder cancer burden among populations aged ≥55 years across 21 regions and 204 countries/territories from 1990 to 2021 using the GBD 2021 dataset. While the GBD database provides a global perspective on risk factors for bladder cancer, it lacks detailed individual-level data. Therefore, this study further integrates NHANES data to comprehensively analyze various risk factors for bladder cancer, including fasting blood glucose and smoking. The study integrates burden distribution mapping, temporal trend evaluation, decomposition analysis, health inequality analysis, predictive analysis, and frontier analysis, providing insights to guide strategic resource allocation and evidence-based policy formulation for high-risk aging populations. This study complies with the TITAN Guidelines 2025 for AI reporting^[14]^.

## Methods

### Data sources

#### GBD database

This study utilized the GBD 2021 dataset, which employs optimized methodologies to evaluate health loss across 204 countries and territories using the latest global epidemiological data^[15]^. The analysis encompasses 371 diseases and injuries, along with 88 risk factors. The GBD 2021 data integration process involves diversified data collection channels, including demographic surveys, household questionnaires, civil registration records, disease registries, healthcare utilization statistics, air quality monitoring, remote sensing imagery, and other health-related databases. From the GBD database, we extracted bladder cancer data for populations aged 55 and older, including metrics such as incidence rates, mortality rates, and DALYs. All data were directly accessible and downloadable via the GBD online data portal(http://ghdx.healthdata.org/gbd-results-tool). This study utilizes the publicly accessible GBD database, which incorporates anonymized datasets, thus exempting the requirement for ethical approval and participant informed consent.

**HIGHLIGHTS**

- Global BC cases surge 113% among elderly (≥55 years) despite crude incidence rate decline (1990–2021).
- Persistent 3.8-fold male-to-female incidence disparity with widening mortality gaps observed.
- Smoking (26.6% DALYs) and high fasting glucose (8.2%) emerge as dual preventable risk drivers.
- Mortality projected to decline 16.4% by 2036, signaling sustained disease control efficacy.
- Central Europe’s rising mortality contrasts with declines in high-income regions.

#### National Health and Nutrition Examination Survey (NHANES) database

The National Health and Nutrition Examination Survey (NHANES), administered by the National Center for Health Statistics (NCHS), assesses the health and nutritional status of U.S. children and adults. This program collects individual-level data through interviews, physical examinations, and laboratory testing. Bladder cancer diagnosis was defined by questionnaire responses in NHANES. Our study utilized eight NHANES cycles (2001–2016), initially including 148 454 participants. After excluding individuals missing fasting glucose measurements, lacking cancer data, having non-bladder malignancies, and lacking other essential variables, the final analytical cohort comprised 14 840 participants. The NHANES protocol was approved by the CDC Research Ethics Review Board (ERB), and all participants provided written informed consent prior to enrollment, confirming full comprehension of study procedures and participant rights.

### Evaluation methods

#### Sociodemographic Index

The Sociodemographic Index (SDI) serves as a composite indicator assessing regional development levels by integrating fertility rates, educational attainment, and income per capita^[16]^. With values scaled between 0 and 1, SDI reflects higher socioeconomic progress as the score increases. Research indicates a correlation between SDI levels and patterns of disease prevalence and mortality. To investigate the relationship between bladder cancer burden and socioeconomic factors, this study categorized countries and regions into five SDI groups: low, low-medium, medium, medium-high, and high.

#### Risk factor analysis

Risk-specific data were extracted from the GBD 2021 database, focusing on bladder cancer risk factors among individuals aged 55 years and older. The study aimed to evaluate attributable mortality rates in this population.

#### Decomposition analysis

We used the Das Gupta decomposition method to decompose the change in the burden of bladder cancer in people aged 55 years or older between 1990 and 2021 into the contributions of aging, population growth, and epidemiologic changes^[17]^. The study performed a global assessment with additional stratification according to SDI categories, allowing for comparative analysis of factor variations across different socioeconomic settings. This approach allows us to dissect the specific contribution of a number of factors to the burden of disease as a way of understanding how demographic and epidemiologic changes affect disease incidence, deaths, and DALYs.

#### Prediction analysis

To predict the disease burden of bladder cancer in people aged 55 years or older from 2022 to 2036, we used the Bayesian age-period-cohort (BAPC) model, which takes age, period, and cohort factors into account as a means of predicting future trends in the burden of disease. The BAPC model is an ideal tool for the long-term prediction of disease burden due to its high degree of time-series data. The BAPC model, with its high adaptability to time- series data and parameter stability, is an ideal tool for long-term prediction of disease burden. The BAPC model has been widely validated in the field of epidemiology by fully integrating multidimensional temporal features and accurately capturing dynamic trends, and is particularly suitable for analyzing complex population health data with age stratification and multiple cohort interactions. This model leverages Bayesian inference implemented via the Integrated Nested Laplace Approximations (INLA) framework. The INLA approach was integrated with the BAPC model to approximate the marginal posterior distributions, effectively circumventing the mixing and convergence challenges inherent in conventional Bayesian methods that rely on Markov chain Monte Carlo (MCMC) sampling techniques^[18,19]^.

#### Health Inequality Analysis

Health Inequality Analysis (HIA) is a statistical technique designed to assess differences in health status between different groups. The methodology focuses on revealing how factors such as socioeconomic status, place of residence, gender, and age interact with health outcomes. In this study, we used two main indicators: the Slope Index of Inequality (SII) and the Concentration Index (CIX), which is a measure of relative health inequality. These tools allow us to analyze the distribution of health inequalities and their trends in bladder cancer from 1990 to 2021, thus effectively quantifying the impact of socioeconomic factors on health status.

#### Joinpoint regression analysis

Joinpoint regression analysis, also known as segmented regression modeling, is a statistical tool used to analyze disease trends over time. The core concept of the model is to use model fitting to divide a long-term trend line into statistically significant trend segments, each of which is described by a continuous straight line. The intersections between these different trend segments are referred to as turning points. We utilized Joinpoint regression analysis to characterize trends in DALY. Results are expressed as annual percentage change (APC), average annual percentage change (AAPC), and 95% confidence intervals (CI) for each age, period, and cohort group. The analysis was conducted using the Joinpoint Regression Program, Version 5.1.0.0 (April 2024), developed by the National Cancer Institute’s Surveillance Research Program.

#### Frontier analysis

This study used frontier analysis techniques to systematically assess the association between bladder cancer disease burden and socio-demographic development level. The methodology is based on the SDI, a comprehensive development measurement system that integrates multidimensional indicators such as per capita income, education level, and fertility rate, and constructs a nonlinear boundary model for determining the theoretically achievable minimum rate of DALYs at a given level of development. By calculating the difference between the observed DALYs and the theoretical boundaries, known as the “effective difference,” the unrealized potential for health improvement in each country at the current level of development is quantified, providing a scientific basis for optimizing resource allocation.

### Statistical analyses

Incidence, deaths, and DALYs rates, along with their corresponding ratios, were the primary indicators used to characterize the burden of bladder cancer in individuals aged over 55 years. According to the GBD algorithm, each rate was reported per 100 000 population, accompanied by a 95% uncertainty interval. The temporal trends in bladder cancer burden among the elderly were analyzed by calculating the EAPC (Estimated Annual Percentage Change). The 95% CI for the EAPC was determined using linear regression modeling. A decreasing trend was inferred if both the EAPC and its 95% CI upper limit were negative, while an increasing trend was indicated if both the EAPC and its 95% CI lower limit were positive. In the NHANES database, continuous variables are expressed as mean ± standard deviation, while categorical variables are presented as counts and percentages. All statistical analyses were conducted using R software (version 4.3.1), with a *P* value <0.05 set as the threshold for statistical significance.

### STROCSS statement

The study followed the guidelines for reporting cohort, cross-sectional, and case–control studies in surgery as outlined by the STROCSS criteria^[20]^.

## Results

### Global trends

Globally, the number of bladder cancer incident cases among individuals aged 55 years and older increased from 226 421.13 (95% UI: 213 091.89 to 236 533.04) in 1990 to 483 233.42 (95% UI: 440 648.27 to 520 931.77) in 2021, representing a 113% rise (95% UI: 99% to 134%) over this period. However, the incidence rate demonstrated a downward trend, with an estimated annual percentage change (EAPC) of −0.22 (95% UI: −0.29 to −0.14) (Table 1). Concurrently, the mortality rate in this population declined from 16.83 per 100 000 (95% UI: 15.57 to 17.72) in 1990 to 14.11 per 100 000 (95% UI: 12.72 to 15.43) in 2021, corresponding to an EAPC of −0.7 (95% UI: −0.77 to −0.63) (Supplementary Digital Content, Table 1, available at: http://links.lww.com/JS9/E918).

**Table 1:** Incidence of bladder cancer in individuals aged 55 and above between 1990 and 2021 at the global and regional level

	Rate per 100 000 (95% UI)					
	1990		2021		1990–2021	
Location	Incident cases	Incident rate	Incident cases	Incident rate	Cases change	EAPC
**Global**	226 421.13 (213 091.89 to 236 533.04)	33.72 (31.74 to 35.23)	483 233.42 (440 648.27 to 520 931.77)	32.52 (29.65 to 35.06)	1.13 (0.99 to 1.34)	−0.22 (−0.29 to −0.14)
**SDI**						
High SDI	114 107.4 (108 349.04 to 117 981.58)	61.2 (58.11 to 63.27)	213 285.8 (192 739.81 to 225 255.42)	61.82 (55.86 to 65.29)	0.87 (0.78 to 0.93)	−0.04 (−0.11 to 0.02)
High middle SDI	71 155.74 (65 737.12 to 75 621.59)	41.24 (38.1 to 43.83)	146 631.94 (133 694.76 to 162 295.9)	42.3 (38.56 to 46.81)	1.06 (0.88 to 1.29)	−0.03 (−0.17 to 0.11)
Middle SDI	24 432.04 (20 598.88 to 27 164.09)	14.08 (11.87 to 15.65)	81 705.93 (70 867.89 to 96 193.28)	17.39 (15.08 to 20.47)	2.34 (1.78 to 3.27)	0.52 (0.44 to 0.59)
Low middle SDI	11 493.36 (9915.76 to 12 963.14)	11.4 (9.84 to 12.86)	30 100.19 (26 414.95 to 39 991.87)	12.49 (10.96 to 16.59)	1.62 (1.16 to 2.52)	0.02 (−0.1 to 0.14)
Low SDI	4945.46 (4129.45 to 5752.56)	13.26 (11.07 to 15.42)	10 896.31 (9478.53 to 12 636.35)	13.28 (11.55 to 15.4)	1.2 (0.87 to 1.63)	−0.15 (−0.23 to −0.07)
**Regions**						
Andean Latin America	337.2 (291.87 to 395.66)	10.05 (8.7 to 11.79)	1236.06 (991.75 to 1543.54)	12.48 (10.01 to 15.58)	2.67 (1.8 to 3.63)	0.7 (0.55 to 0.86)
Australasia	2175.51 (2028.18 to 2312.49)	55.22 (51.48 to 58.7)	3849.15 (3453.77 to 4197.48)	43.57 (39.1 to 47.51)	0.77 (0.6 to 0.96)	−0.94 (−1.07 to −0.81)
Caribbean	1011.27 (953.83 to 1066.14)	23.46 (22.13 to 24.74)	2507.55 (2183.43 to 2784.44)	27.08 (23.58 to 30.07)	1.48 (1.18 to 1.8)	0.72 (0.59 to 0.85)
Central Asia	1340.44 (1195.64 to 1503.25)	16.76 (14.95 to 18.8)	2349.64 (2065.28 to 2626.63)	16.15 (14.19 to 18.05)	0.75 (0.52 to 1.05)	−0.35 (−0.61 to −0.08)
Central Europe	10 830.2 (10 362.98 to 11 296.42)	40.84 (39.08 to 42.6)	25 485.36 (23 292.98 to 27 497.21)	68.83 (62.91 to 74.26)	1.35 (1.18 to 1.54)	1.57 (1.43 to 1.71)
Central Latin America	1472.61 (1412.35 to 1520.9)	10.85 (10.41 to 11.21)	5030.46 (4457.01 to 5577.92)	11.76 (10.42 to 13.04)	2.42 (2.05 to 2.79)	0.1 (−0.02 to 0.22)
Central Sub-Saharan Africa	522.14 (423.84 to 658.94)	13.89 (11.27 to 17.52)	1260.76 (974.5 to 1626.45)	13.97 (10.8 to 18.02)	1.41 (0.84 to 2.14)	−0.08 (−0.2 to 0.04)
East Asia	29 208.96 (21 811.33 to 34 024.3)	19.61 (14.64 to 22.84)	94 186.86 (75 398.3 to 120 279.89)	24.02 (19.23 to 30.67)	2.22 (1.43 to 3.65)	0.44 (0.33 to 0.54)
Eastern Europe	13 807.9 (13 055.2 to 14 835.65)	28.24 (26.7 to 30.34)	20 817.7 (18 783.77 to 22 755.61)	33.53 (30.26 to 36.66)	0.51 (0.35 to 0.67)	0.15 (−0.12 to 0.43)
Eastern Sub-Saharan Africa	1911.73 (1607.53 to 2297.31)	15.71 (13.21 to 18.88)	4116.03 (3400.9 to 5061.28)	15.22 (12.58 to 18.72)	1.15 (0.77 to 1.57)	−0.28 (−0.34 to −0.21)
High-income Asia Pacific	11 237.43 (10 537.04 to 11 773.99)	32.14 (30.13 to 33.67)	33 087.85 (28 634.64 to 36 141.58)	46.93 (40.61 to 51.26)	1.94 (1.69 to 2.15)	1.31 (1.23 to 1.39)
High-income North America	45 030.35 (42 476.22 to 46 743.2)	77.73 (73.33 to 80.69)	86 478.65 (79 123.19 to 90 824.65)	76.85 (70.31 to 80.71)	0.92 (0.85 to 0.99)	−0.2 (−0.35 to −0.05)
North Africa and Middle East	8672.52 (6965.71 to 10 313.7)	30.68 (24.65 to 36.49)	29 467.64 (24 961.44 to 35 821.96)	38.65 (32.74 to 46.99)	2.4 (1.65 to 3.47)	0.63 (0.55 to 0.71)
Oceania	34.18 (23.15 to 46.44)	7.11 (4.81 to 9.65)	109.74 (71.05 to 148.39)	8.89 (5.76 to 12.02)	2.21 (1.54 to 3.12)	0.8 (0.76 to 0.85)
South Asia	8406.29 (6842.66 to 9750.24)	8.85 (7.21 to 10.27)	26 074.69 (22 709.69 to 31 768.79)	10.5 (9.15 to 12.79)	2.1 (1.5 to 2.94)	0.27 (0.13 to 0.41)
Southeast Asia	3599.78 (3155.69 to 4100.28)	8.5 (7.45 to 9.68)	12 820.69 (10 854.06 to 16 043)	11.19 (9.48 to 14)	2.56 (1.94 to 3.33)	0.68 (0.6 to 0.77)
Southern Latin America	3047.2 (2842.56 to 3228.23)	38.47 (35.88 to 40.75)	4713.15 (4347.9 to 5051.27)	32.03 (29.55 to 34.32)	0.55 (0.42 to 0.68)	−0.51 (−0.63 to −0.39)
Southern Sub-Saharan Africa	708.25 (568.99 to 874.9)	16.01 (12.86 to 19.77)	1802.43 (1604.85 to 1998.61)	18.51 (16.48 to 20.53)	1.54 (1.11 to 2.09)	0.33 (0.1 to 0.56)
Tropical Latin America	2594.99 (2451.41 to 2705.31)	17.14 (16.19 to 17.87)	8861.51 (8128.38 to 9387.8)	20 (18.35 to 21.19)	2.41 (2.21 to 2.62)	0.48 (0.38 to 0.57)
Western Europe	79 041.73 (75 179.01 to 81 683.62)	81.39 (77.41 to 84.11)	115 751.15 (104 469.87 to 123 621.51)	77.62 (70.05 to 82.89)	0.46 (0.38 to 0.54)	−0.13 (−0.2 to −0.05)
Western Sub-Saharan Africa	1430.46 (1219.25 to 1645.94)	9.91 (8.45 to 11.4)	3226.35 (2702.51 to 3853.3)	10.04 (8.41 to 11.99)	1.26 (0.85 to 1.75)	0.01 (−0.06 to 0.08)

EAPC, estimated annual percentage change; SDI, Sociodemographic Index; UI, uncertainty interval.

Additionally, the DALYs rate exhibited a significant decreasing trend, dropping from 337.59 (95% UI: 310.76 to 356.98) in 1990 to 257.76 (95% UI: 237.13 to 281.81) in 2021, with an EAPC of −1.04 (95% UI: −1.11 to −0.97). The total DALYs cases rose from 2 266 686.97 (95% UI: 2 086 495.83 to 2 396 854.59) in 1990 to 3 830 290.68 (95% UI: 3 523 747.89 to 4 187 719.83) in 2021, reflecting a 69% increase (Supplementary Digital Content, Table 2, available at: http://links.lww.com/JS9/E919). This phenomenon may imply that under the pressure of a growing patient base, longer survival and a significant reduction in the burden of disability for more elderly patients through early diagnosis (e.g., widespread screening for urinary tumor markers) and targeted therapeutic advances (e.g., the use of immunotherapies) is centrally attributable to multinational tobacco control policies and investment in precision medicine in high-income countries^[21]^.

### Regional trends across 21 geographic areas

**Figure 1.**
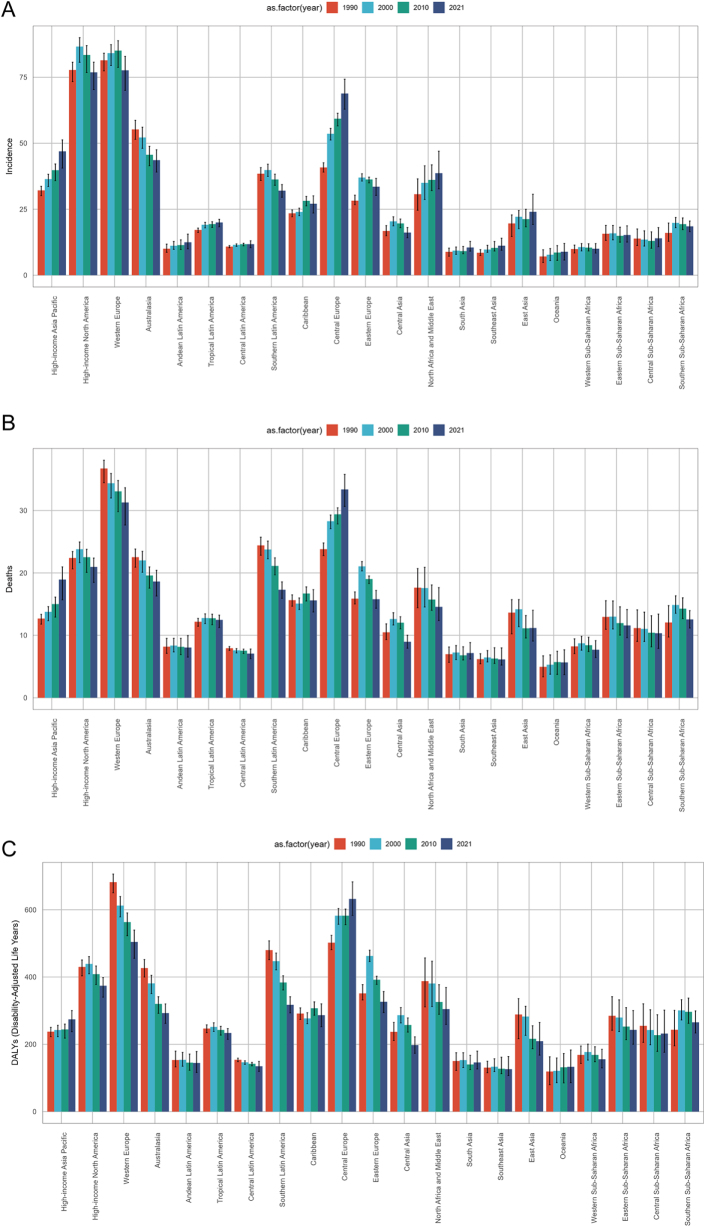
Global bladder cancer (A) incidence, (B) mortality, and (C) DALYs rates among populations aged ≥55 years across GBD regions in 1990, 2000, 2010, and 2021.

Under the GBD regional classification system, we analyzed temporal changes and disparities in bladder cancer incidence, deaths, and DALYs rates among individuals aged ≥55 years across 21 geographic regions during 1990, 2000, 2010, and 2021 (Fig. 1A). High-income North America and Western Europe exhibited the most concerning epidemiological profiles. In 2021, bladder cancer incidence rates in these regions reached 76.85 (95% UI: 70.31 to 80.71) and 77.62 (95% UI: 70.05 to 82.89) per 100 000 population, respectively. Both regions experienced peak incidence around 2000–2010, yet demonstrated declining trends by 2021 compared to 1990 levels, with EAPC values of −0.2 (95% UI: −0.3 to −0.05) and −0.13 (95% UI: −0.2 to −0.05) (Supplementary Digital Content, Figure 1, available at: http://links.lww.com/JS9/E917).

**Figure 2.**
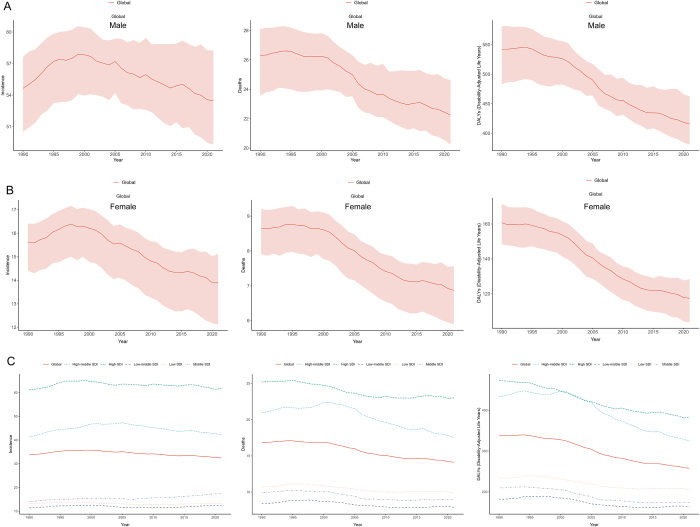
Global trends in (A) incidence, mortality, and DALYs rates of bladder cancer among males aged 55 years and older, and (B) incidence, mortality, and DALYs rates among females aged 55 years and older from 1990 to 2021. From 1990 to 2021, (C) global trends in incidence, mortality, and DALYs rates across five SDI regions.

In contrast, Oceania consistently maintained favorable indicators, recording the lowest 2021 incidence rate at 2.21 (95% UI: 1.54 to 3.12) per 100 000 (Fig. 1A and Table 1). Deaths and DALYs rates showed marked regional heterogeneity: Western Europe and Central Europe displayed the highest burden, yet diverged in trends. While Western Europe demonstrated decreasing rates, Central Europe exhibited upward trajectories for both mortality (EAPC 0.97; 95% UI: 0.86 to 1.08) and DALYs (EAPC 0.58; 95% UI: 0.47 to 0.69) (Fig. 1B and C; Supplementary Digital Content, Tables 1–2, available at: http://links.lww.com/JS9/E918; http://links.lww.com/JS9/E919). This divergence persisted despite declining trends observed in most other regions, highlighting Central Europe’s unique epidemiological transition pattern.

### Gender- and SDI-specific global trends

Between 1990 and 2021, the bladder cancer burden in individuals aged ≥55 years declined across both sexes, though significant gender disparities persisted. Males consistently demonstrated higher burden severity (Fig. 2A and B). Male incidence rates decreased from 54.64 (95% UI: 50.5 to 57.63) to 53.47 (95% UI: 49.27 to 58.25), mortality rates decreased from 26.27 (95% UI: 23.55 to 28.11) to 22.25 (95% UI: 20.23 to 24.62), and DALYs rates decreased from 541.99 (95% UI: 482.98 to 581.29) to 415.54 (95% UI: 380.62 to 462.47). Female metrics showed greater improvement: incidence rates decreased from 15.62 (95% UI: 14.4 to 16.41) to 13.89 (95% UI: 12.12 to 15.12), mortality rates decreased from 8.66 (95% UI: 7.9 to 9.2) to 6.87 (95% UI: 5.89 to 7.56), and DALYs rates fell from 160.72 (95% UI: 148.27 to 171.56) to 117.42 (95% UI: 103.64 to 128.29) (Supplementary Digital Content, Table 3, available at: http://links.lww.com/JS9/E920).

**Figure 3.**
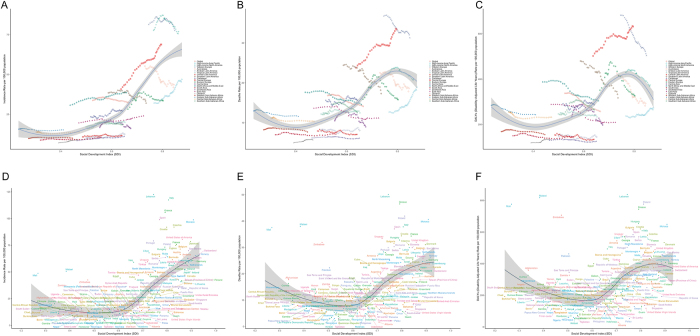
Global trends in (A) incidence rates, (B) mortality rates, and (C) DALYs rates of bladder cancer among individuals aged 55 years and older across 21 geographical regions worldwide from 1990 to 2021. Global trends in (D) incidence rates, (E) mortality rates, and (F) DALYs rates of bladder cancer among individuals aged 55 years and older across 204 countries or territories worldwide from 1990 to 2021.

Notably, we observed declining overall disease burden across most SDI regions, except for Middle SDI and Low-middle SDI regions which showed slight increases in incidence rates with EAPC values of 0.52 (95% UI: 0.44 to 0.59) and 0.02 (95% UI: −0.1 to 0.14), respectively (Fig. 2A). The most substantial incidence rate decline occurred in Low SDI regions (EAPC: −0.15; 95% UI: −0.23 to −0.07) (Table 1; Supplementary Digital Content, Figure 1, available at: http://links.lww.com/JS9/E917). Regarding mortality rates and DALYs rates, High-middle SDI regions demonstrated the most pronounced reductions. In contrast, Low SDI regions exhibited the smallest declines (Fig. 2C; Supplementary Digital Content, Figure 1, available at: http://links.lww.com/JS9/E917). Specifically, High-middle SDI regions achieved EAPC values of −0.73 (95% UI: −0.87 to −0.58) for mortality rates and −0.78 (95% UI: −0.82 to −0.73) for DALYs rates (Supplementary Digital Content, Tables 1–2, available at: http://links.lww.com/JS9/E918; http://links.lww.com/JS9/E919; Fig. 3A–C). These differential trends highlight the complex interplay between socioeconomic development and health outcome improvements.

Bladder cancer incidence rates among individuals aged ≥55 years exhibited a positive correlation with SDI levels (Fig. 3A). For mortality rates and DALYs rates, both metrics demonstrated a U-shaped association with SDI: continuous declines occurred until SDI reached ~0.4, peaked between SDI 0.4–0.8, followed by sustained reductions at higher SDI levels (Fig. 3B and C). Regionally, 2021 data revealed Western Europe (SDI = 0.85) had the highest incidence rate and mortality rate, while Central Europe (SDI = 0.77) showed the highest DALYs rate. Conversely, Oceania (SDI = 0.47) recorded the lowest incidence and mortality rates, with Southeast Asia (SDI = 0.69) exhibiting the minimal DALYs rate (Fig. 3A and B).

Globally, bladder cancer cases in 2021 were significantly higher in males than females. Both sexes shared peak incidence and DALYs in the 70–74 age group, while mortality peaked later at 80–84 years. Notably, gender disparities diminished substantially in the ≥95 age group (Supplementary Digital Content, Figure 2, available at: http://links.lww.com/JS9/E917).

### National trends

**Figure 4.**
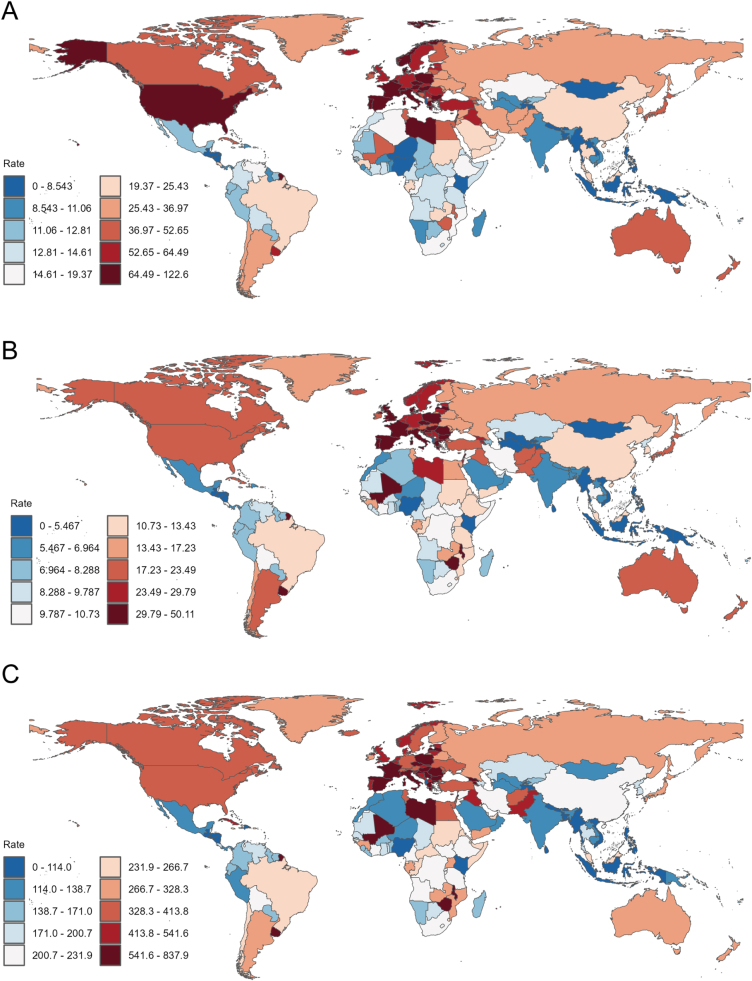
(A) Incidence rates, (B) mortality rates, and (C) DALYs rates of bladder cancer among individuals aged 55 years and older across 204 countries and territories in 2021.

Lebanon (SDI = 0.74) and Italy (SDI = 0.81) exhibited the highest incidence rates at 122.56 (95% UI: 94.2 to 159.3) and 117.41 (95% UI: 104.47 to 127.05) per 100 000 population, respectively (Figs. 3D and 4A). Greece and Spain followed with incidence rates of 105.75 (95% UI: 96.25 to 114.18) and 98.98 (95% UI: 88.37 to 108.92). Conversely, Albania and Kiribati recorded the lowest incidence rates at 3.02 (95% UI: 2.28 to 4.11) and 3.03 (95% UI: 2.27 to 3.83) (Supplementary Digital Content, Table 4, available at: http://links.lww.com/JS9/E921).

For mortality rates, Lebanon (SDI = 0.74) showed the highest value at 50.11 (95% UI: 38.13 to 63.92), while Albania (SDI = 0.71) had the lowest at 1.64 (95% UI: 1.22 to 2.27) (Figs. 3E and 4B; Supplementary Digital Content, Table 5, available at: http://links.lww.com/JS9/E922). Lebanon also displayed the highest DALYs rate (837.91; 95% UI: 653.91 to 1059.77) (Figs. 3F and 4C; Supplementary Digital Content, Table 6, available at: http://links.lww.com/JS9/E923).

China accounted for the largest absolute burden, with 90 134.81 incident cases (95% UI: 71 265.12 to 116 289.04), 42 045.62 deaths (95% UI: 33 941.81 to 53 213.82), and 787 232.86 DALYs (95% UI: 624 579.04 to 1 003 913.26) (Supplementary Digital Content, Figure 3A–C, available at: http://links.lww.com/JS9/E917; Supplementary Digital Content, Tables 4–6, available at: http://links.lww.com/JS9/E923; http://links.lww.com/JS9/E923).

Mongolia demonstrated the most pronounced incidence rate decline (EAPC: −1.99), while Cabo Verde showed the sharpest increase (EAPC: +5.54). For mortality rates, Bahrain had the largest reduction (EAPC: −3.32), contrasting with Cabo Verde’s significant rise (EAPC: +4.91) (Supplementary Digital Content, Figure 4, available at; http://links.lww.com/JS9/E917; Supplementary Digital Content, Tables 4–6, available at: http://links.lww.com/JS9/E923; http://links.lww.com/JS9/E923).

### Risk factor attribution patterns

**Figure 5.**
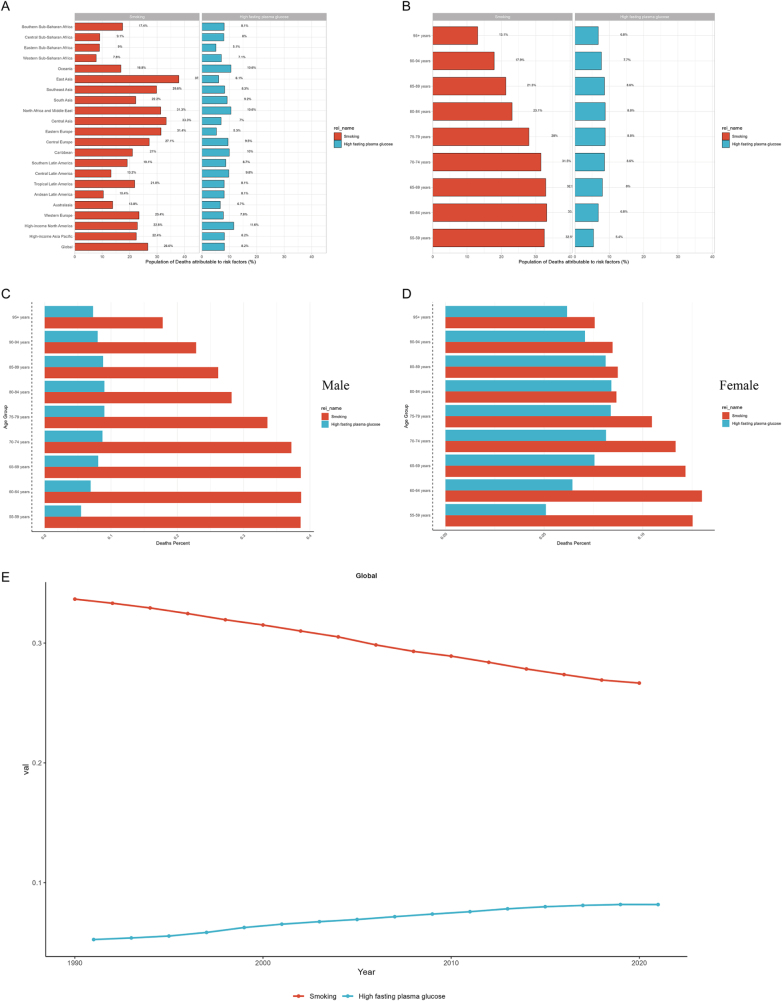
(A) Percentage of bladder cancer deaths attributable to each risk factor across 21 GBD regions. (B) Percentage of bladder cancer deaths attributable to each risk factor among individuals aged 55 years and older across age groups globally. (C) Percentage of bladder cancer deaths attributable to each risk factor among male individuals aged 55 years and older across age groups globally. (D) Percentage of bladder cancer deaths attributable to each risk factor among female individuals aged 55 years and older across age groups globally. (E) Trends in the percentage of bladder cancer deaths attributable to each risk factor from 1990 to 2021.

Two primary risk factors contributed to bladder cancer mortality among individuals aged ≥55 years globally: smoking (attributable fraction: 26.6%) and high fasting plasma glucose (8.2%). Geographically, smoking exhibited the strongest impact in East Asia and the weakest in Western Sub-Saharan Africa. Meanwhile, high fasting plasma glucose showed the highest attribution in High-income North America and the lowest in Eastern Sub-Saharan Africa (Fig. 5A). Age-stratified analysis revealed peak smoking-attributable fractions in the 60–64 age group, whereas high fasting plasma glucose reached maximal impact in the 80–84 age group (Fig. 5B). Gender disparities were evident: smoking dominated mortality attribution in males, while females exhibited a substantially elevated proportion of deaths linked to high fasting plasma glucose, though smoking remained their primary risk factor (Fig. 5C and D). Temporal trends from 2019 to 2021 demonstrated a progressive decline in smoking-attributable fractions, contrasted by a concurrent rise in contributions from high fasting plasma glucose (Fig. 5E). This pattern reflects both the success of global tobacco control initiatives and emerging challenges posed by metabolic risk factors associated with improving living standards.

### 3.6 Baseline characteristics in NHANES Population

The NHANES dataset included 14 840 participants, comprising 35 bladder cancer cases and 14 805 controls. Based on risk analysis findings from the GBD database, we incorporated relevant indicators from NHANES for further investigation. Bladder cancer patients were predominantly male, older, Non-Hispanic White, married, hypertensive, with higher fasting glucose levels, and were smokers. The mean fasting glucose was 5.95 ± 1.98 mmol/L in controls versus 6.74 ± 3.33 mmol/L in bladder cancer cases (Supplementary Digital Content, Table 7, available at: http://links.lww.com/JS9/E924). Supplementary Digital Content, Table 8 (available at: http://links.lww.com/JS9/E925) details baseline characteristics by fasting glucose quartiles (Q1: 1.17–5.107; Q2: 5.107–5.495; Q3: 5.495–6.051; Q4: 6.051–32.418 mmol/L). Compared to the lowest quartile (Q1), individuals in the highest quartile (Q4) were more frequently male, older, smokers, and had comorbid hypertension. We have found that the incidence of bladder cancer tends to increase as fasting blood glucose increases. Based on this, we further found that fasting blood glucose has excellent diagnostic ability for bladder cancer by constructing a ROC curve with an area under the curve (AUC) of 0.650 (Supplementary Digital Content, Figure 5, available at: http://links.lww.com/JS9/E917).

### Subgroup analysis by fasting glucose in NHANES

Supplementary Digital Content, Figure 6 (available at: http://links.lww.com/JS9/E917) presents subgroup-specific results. Significantly elevated risks were observed in: the overall population (OR_Q4vs1_ = 3.483; 95% CI: 1.145–10.591; *P* = 0.028)

married or living with partner individuals (OR_Q4vs1_ = 5.536; 95% CI: 1.238–24.761; *P* = 0.025), smokers (OR_Q4vs1_ = 7.754; 95% CI: 0.992–60.640; *P* = 0.048), alcohol consumers (OR_Q4vs1_ = 6.625; 95% CI: 1.494–29.370; *P* = 0.013), and non-hypertensive participants (OR_Q4vs1_ = 10.784; 95% CI: 1.298–89.625; *P* = 0.028). These findings indicate the association between fasting glucose and bladder cancer incidence appears confined to specific subgroups.

### Decomposition analysis of bladder cancer burden in individuals aged ≥55 years

**Figure 6.**
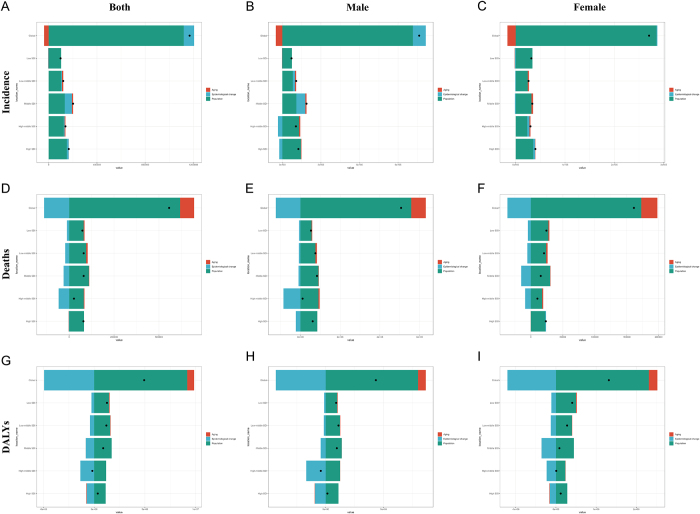
Decomposition analysis of bladder cancer burden among individuals aged 55 years and older from 1990 to 2021. (A) Decomposition analysis of changes in incident cases of bladder cancer for both sexes. (B) Decomposition analysis of changes in incident cases of bladder cancer among males. (C) Decomposition analysis of changes in incident cases of bladder cancer among females. (D) Decomposition analysis of changes in death cases of bladder cancer for both sexes. (E) Decomposition analysis of changes in death cases of bladder cancer among males. (F) Decomposition analysis of changes in death cases of bladder cancer among females. (G) Decomposition analysis of changes in DALYs of bladder cancer for both sexes. (H) Decomposition analysis of changes in DALYs of bladder cancer among males. (I) Decomposition analysis of changes in DALYs of bladder cancer among females.

The global burden of bladder cancer in this population was driven by three factors: aging, population growth, and epidemiological changes. Aging contributed −3.22% to global incident cases but led to increases of 14.02% in deaths and 13.79% in DALYs. Population growth resulted in rises of 95.85% in incident cases, 110.98% in deaths, and 186.13% in DALYs. Epidemiological changes caused a 7.36% increase in incident cases, a 24.99% reduction in deaths, and a 99.92% reduction in DALYs. Population aging contributed to a decrease in global incident cases, while driving increases in deaths and DALYs. Among these factors, population growth exhibited the most substantial influence, consistently elevating incident cases, deaths, and DALYs. Notably, epidemiological changes elevated incidence rates globally but reduced rates in males from High-middle SDI and High SDI regions. For absolute burden metrics (incident cases and DALYs), epidemiological changes consistently decreased impacts across regions and sexes. These results highlight significant geographic and gender disparities in burden drivers (Fig. 6; Supplementary Digital Content, Table 9, available at: http://links.lww.com/JS9/E926).

### Predictive analysis

**Figure 7.**
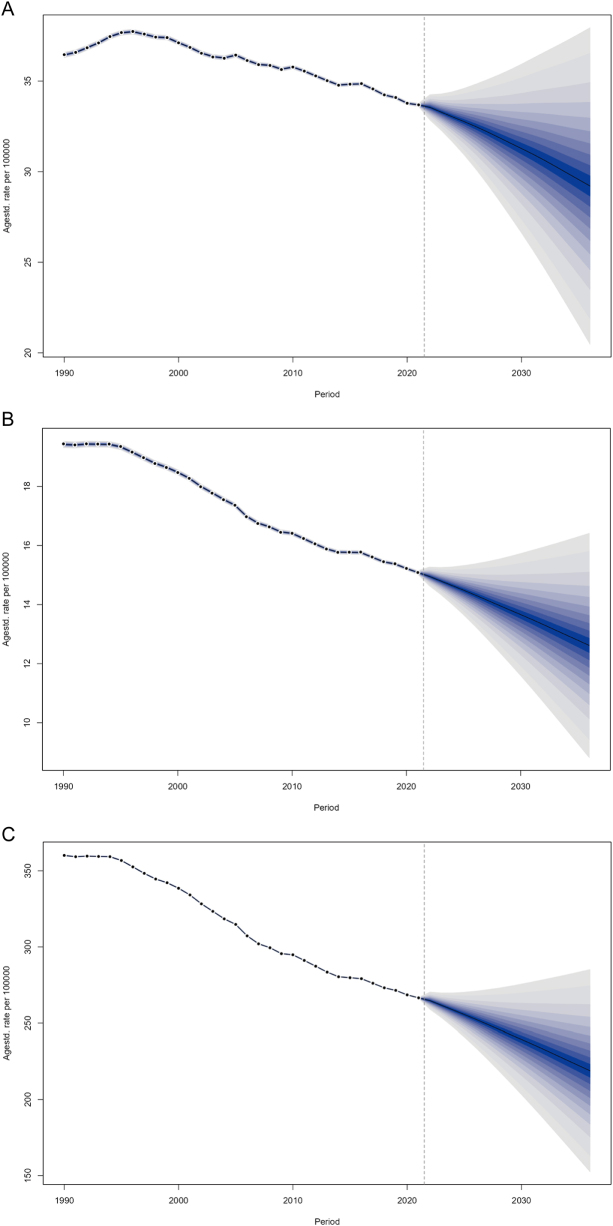
(A) Predicted global incidence rate of bladder cancer in 2036. (B) Predicted global mortality rate of bladder cancer in 2036. (C) Predicted global DALYs rate of bladder cancer in 2036.

Using the BAPC model, we projected global trends in bladder cancer incidence rate, mortality rate, and DALYs rate among individuals aged ≥55 years from 2022 to 2036. By 2036, the incidence rate is projected to reach 29.21 per 100 000, reflecting a 13.3% decrease compared to 2021 (29.21 vs. 33.68). The mortality rate is expected to decline to 12.62 per 100 000 (16.4% reduction from 2021’s 15.09), while the DALYs rate is forecasted to drop to 218.83 per 100 000 (17.9% decrease from 2021’s 266.53) (Fig. 7; Supplementary Digital Content, Table 10, available at: http://links.lww.com/JS9/E927). We further projected bladder cancer incidence, deaths, and DALYs across different age groups (≥55 years) globally from 2022 to 2036. The analysis revealed that the predicted decline in incidence rates, mortality rates, and DALYs rates progressively moderated with increasing age (Supplementary Digital Content, Figure 7, available at: http://links.lww.com/JS9/E917; Supplementary Digital Content, Table 11, available at: http://links.lww.com/JS9/E928). Despite projected global declines in bladder cancer burden, our age-stratified analysis reveals a persistent negative impact of aging, manifested through progressively attenuated reductions in incidence, mortality, and DALY rates with advancing age.

### Health inequality analysis

**Figure 8.**
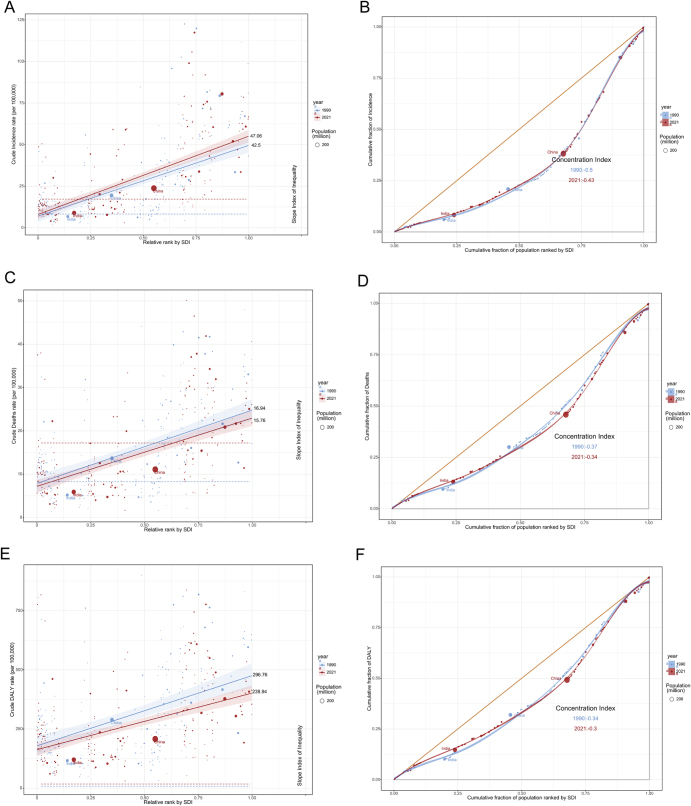
Health inequality analysis of global bladder cancer burden among individuals aged 55+ in 1990 and 2021. (A) SII analysis of bladder cancer incidence and (B) CIX analysis of incidence. (C) SII analysis of bladder cancer mortality and (D) CIX analysis of mortality. (E) SII analysis of bladder cancer DALYs and (F) CIX analysis of DALYs.

To examine socioeconomic disparities in bladder cancer burden among individuals aged ≥55 years, we analyzed health inequalities using the Slope Index of Inequality (SII) and Concentration Index (CIX) across SDI levels from 1990 to 2021. In 2021, the SII values for bladder cancer incidence rate, mortality rate, and DALYs rate were 47.06, 15.76, and 238.94, respectively. These values were 42.5, 16.94, and 296.76 in 1990. For relative inequality, the 2021 concentration indices were −0.43 (incidence), −0.34 (mortality), and −0.3 (DALYs). The slight decrease in concentration indices suggests a gradual reduction in relative inequality over this period (Fig. 8).

### Joinpoint analysis

**Figure 9.**
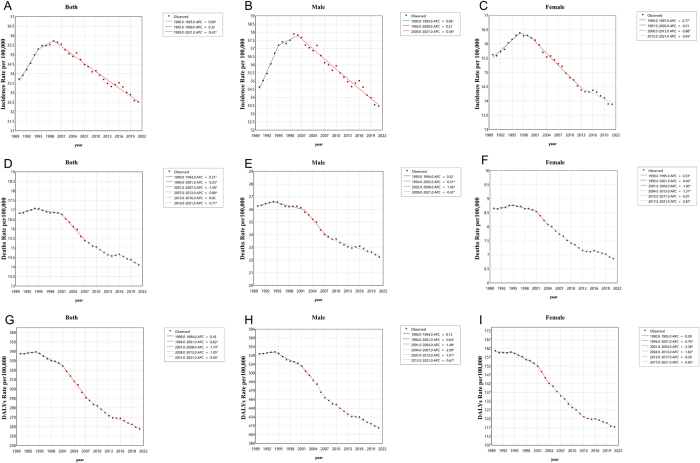
Joinpoint analysis of bladder cancer burden among individuals aged 55 years and older from 1990 to 2021. (A) Joinpoint analysis of global incidence trends in both sexes. (B) Joinpoint analysis of global incidence trends in males. (C) Joinpoint analysis of global incidence trends in females. (D) Joinpoint analysis of global mortality trends in both sexes. (E) Joinpoint analysis of global mortality trends in males. (F) Joinpoint analysis of global mortality trends in females. (G) Joinpoint analysis of global DALY rate trends in both sexes. (H) Joinpoint analysis of global DALY rate trends in males. (I) Joinpoint analysis of global DALY rate trends in females.

Using Joinpoint regression, we analyzed temporal trends in bladder cancer burden among individuals aged ≥55 years. Globally, the incidence rate showed an upward trend from 1990 to 1995 (Annual Percent Change [APC] = +0.96), followed by a decelerated increase from 1995 to 1999 (APC = +0.32), and transitioned to a sustained decline from 1999 to 2021 (APC = −0.42). For mortality rate, an initial rise was observed from 1990 to 1994 (APC = +0.37), succeeded by a gradual decline from 1994 to 2001 (APC = −0.23), an accelerated reduction from 2001 to 2007 (APC = −1.39), a moderated decline from 2007 to 2013 (APC = −0.89), nonsignificant changes from 2013 to 2016, and resumed downward trends from 2016 to 2021 (APC = −0.71). The DALYs rate exhibited nonsignificant fluctuations from 1990 to 1994, followed by a declining phase from 1994 to 2001 (APC = −0.62), and continued reductions at progressively diminishing rates from 2001 to 2021. These patterns remained consistent between males and females globally (Fig. 9; Supplementary Digital Content, Table 12, available at: http://links.lww.com/JS9/E929).

### Frontier analysis of bladder cancer burden

**Figure 10.**
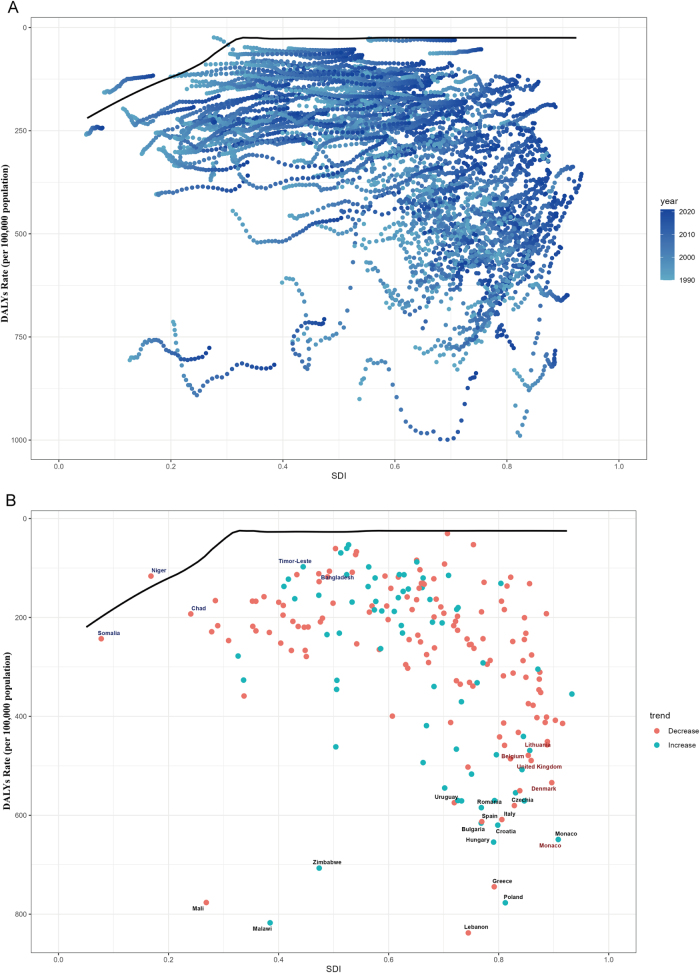
(A) Frontier analysis of SDI and BC-related DALY rates (1990–2021). (B) Frontier analysis of SDI and BC-related DALY rates (2021).

By comparing health outcomes against optimal frontiers across countries and territories from 1990 to 2021, we assessed potential improvement spaces in alleviating bladder cancer burden among individuals aged ≥55 years. Figure 10 and Supplementary Digital Content, Table 13 (available at: http://links.lww.com/JS9/E930) present the 2021 DALYs rates and effective gaps (observed DALYs minus frontier-predicted minimums) by development level. Countries and territories with the largest effective difference included Lebanon, Malawi, Poland, Greece, and Zimbabwe. Conversely, Niger, Somalia, Albania, Kiribati, and Nigeria exhibited the smallest effective difference. The analysis revealed paradoxically stronger capacity for burden control in some low-SDI regions compared to higher-SDI counterparts. Notably, several high/middle-SDI countries – including Poland, Greece, Hungary, Monaco, and Croatia – demonstrated substantial room for improvement despite their advanced development levels.

## Discussion

### Clinical significance and demographic shifts in bladder cancer burden

Bladder cancer represents a predominant malignancy within the genitourinary tract, with the muscle-invasive subtype (MIBC) demonstrating an elevated propensity for metastatic progression compared to non-invasive variants^[22,23]^. The majority of patients require long-term cystoscopic monitoring and multiple therapies, which not only diminish their health-related quality of life but also impose a substantial healthcare burden^[21]^. Consequently, bladder cancer has become one of the most costly malignancies to treat^[24]^. Over the past 50 years, Western population age structures have shifted from a pyramid to a column-like pattern, marked by accelerated aging, rising life expectancy (e.g., 78.7 to 82.5 years in Italy by 2045), and a projected surge in the 85+ age group (from 3.7 million in 1996 to 18.2 million in 2050 in the U.S.), with seniors expected to comprise 62% of the population by mid-century^[25]^. Epidemiological evidence confirms that incidence rates and advancing age serve as independent predictors of oncogenesis across malignancies, a pattern consistently observed in bladder cancer^[26,27]^. Therefore, systematic evaluation of epidemiological characteristics and risk stratification in high-risk bladder cancer subgroups serves as a cornerstone for public health policymakers and clinical teams to formulate evidence-based prevention protocols, optimize targeted screening strategies, and develop personalized treatment pathways.

### Key epidemiological patterns and targeted intervention strategies

This study analyzes global, regional, and national bladder cancer data among individuals aged ≥55 years from 1990 to 2021, leveraging the GBD 2021 dataset. The core analysis incorporates incidence, deaths, and disability-adjusted life years (DALYs), while advanced methodologies explore temporal trends, decomposition analysis, health inequalities, future predictions, Joinpoint regression, and frontier analysis. In 2021, global bladder cancer cases showed a marked male predominance. This disparity is primarily mediated through the higher smoking prevalence among males – the most established risk factor for bladder carcinogenesis. Consequently, sustained, population-level tobacco control initiatives represent a pivotal strategy to attenuate the projected bladder cancer incidence and mortality rates over decadal timelines^[28]^. Both sexes exhibited peak incidence and DALYs rates in the 70-74 age group, whereas mortality peaked later, in the 80-84 age group. This highlight pronounced gender and age-specific disparities in bladder cancer burden within this population. This underscores the necessity to intensify health education, elevate disease prevention awareness, and implement targeted screening programs with regular check-ups for high-risk age cohorts, thereby enabling early detection and intervention of bladder cancer to optimize therapeutic outcomes and enhance patients’ quality of life.

### Socioeconomic determinants of divergent regional trajectories

The current overview of the global burden of cancer in major high-income countries shows that mortality from most cancers has declined over the past few decades^[29]^. Bladder cancer mortality rates have experienced the most significant decline in certain developed countries and nations undergoing rapid economic transitions^[30]^. Our study reveals that while global bladder cancer incidence in populations aged ≥55 years exhibits an overall downward trajectory, low-middle SDI and middle SDI regions show persistent upward trends. For mortality and DALY rates in this population, we observed noteworthy findings: although both metrics demonstrate declining patterns, the rate of reduction in lower-SDI regions remains slower compared to high-SDI counterparts. Tobacco control policies in developed nations (e.g., increased taxation, advertising bans) are strongly associated with declining smoking rates, while low- and middle-income countries continue to face targeted market expansion strategies by tobacco industries. These disparities in policy implementation may partially explain the international heterogeneity in bladder cancer burden^[31]^. In addition to smoking, our findings indicate that high fasting plasma glucose (HFPG) independently contributes to bladder cancer mortality. Notably, the risk magnitude of HFPG in female populations is comparable to that of smoking. Female patients exhibit a distinct “metabolic risk transition” – particularly in the advanced-age cohort over 80 years, where hyperglycemia’s mortality contribution nearly equals that of smoking. This sex-specific divergence necessitates a pivotal strategic shift: bladder cancer prevention in elderly women should transition from traditional tobacco-centered interventions to integrated management of metabolic syndrome. Furthermore, the contribution of HFPG to bladder cancer mortality has shown a progressively increasing trend over time. This upward trend may be attributed to rapid urbanization and industrialization in recent decades, which have significantly altered lifestyle patterns – such as increased sedentary behavior and imbalanced diets – ultimately leading to a rapid rise in blood glucose levels^[32]^. East Asia bears the world’s highest smoking-attributable mortality burden, embodying the profound influence of industrialization and deeply entrenched tobacco culture. Conversely, high-income North America emerges as the epicenter of hyperglycemia-related risk, pointing to systemic failures in managing processed food dependence and metabolic health. In stark contrast, sub-Saharan Africa exhibits a unique “dual-low barrier” – with Eastern Africa demonstrating substantially lower attributable fractions for both smoking and hyperglycemia – likely stemming from poverty-driven constraints on tobacco access and traditional diets characterized by low-glycemic-index staples. Several studies have reported that cancer patients with both diabetes and elevated fasting plasma glucose (FPG) levels may experience reduced efficacy of neoadjuvant chemotherapy^[33,34]^. Concurrently, high FPG levels can induce insulin resistance, promoting tumor growth and ultimately leading to poor prognosis in cancer patients^[35]^. In this context, middle-aged and elderly bladder cancer patients may face a higher risk of comorbid diabetes. Therefore, it is crucial to emphasize the importance of healthy dietary habits and regular physical activity for this population to mitigate the growing burden of elevated FPG on bladder cancer outcomes.

### Aging-driven burden projections and mitigation frameworks

BAPC projections indicate declining trends in bladder cancer incidence, mortality, and DALYs rates among individuals aged ≥55 years. However, age-stratified analyses revealed an intriguing attenuation of these projected declines with advancing age, a phenomenon potentially attributable to population aging, global demographic expansion, and emergence of novel risk factors^[36]^. Strengthening age-specific preventive measures, promoting healthy lifestyles, and enhancing early detection programs for the elderly could help mitigate the disease’s burden. Additionally, policies targeting risk factors like smoking and occupational exposures, coupled with improved healthcare access for aging populations, may further reduce bladder cancer’s impact in older age groups.

### Health inequality mechanisms and contextualized policy solutions

Despite substantial advancements in healthcare, global health inequalities persist as a pervasive and enduring issue, remaining a formidable public health challenge^[37,38]^. Our health inequality analysis reveals that socioeconomic disparities persist as fundamental drivers of bladder cancer burden differentials across development strata. While marginal improvements in relative inequality occurred over the past three decades – evident through converging concentration indices – absolute disparities remain entrenched, particularly in high-SDI regions exhibiting elevated disease incidence. This paradox underscores how socioeconomic progress mediates bladder cancer epidemiology through divergent pathways: higher development levels facilitate advanced diagnostic capabilities and tobacco control policies that reduce mortality, yet simultaneously increase population exposure to modifiable risks like smoking and hyperglycemia through sedentary lifestyles and dietary shifts. Consequently, nations with identical SDI scores manifest heterogeneous burden patterns, as demonstrated by Lebanon’s outlier status in our frontier analysis. To disrupt this complex nexus, future interventions must integrate two-pronged strategies: (1) scaling resource-appropriate early detection in low-SDI regions where late-stage diagnosis exacerbates mortality, and (2) deploying targeted metabolic risk mitigation in high-SDI settings where incidence remains disproportionately elevated despite declining trends.

### Public health implications and research limitations

This study provides critical insights for enhancing public health strategies and clinical management of bladder cancer in elderly populations. We analyzed the trends in bladder cancer incidence, deaths, and DALYs rates over time and across regions in the elderly. We also examined the risk factors for bladder cancer mortality and their changing trends. Based on the results of the GBD risk analysis, we further integrated data from the NHANES database to validate at the individual level that elevated fasting blood glucose increases the risk of bladder cancer. Through frontier analysis, we found that some high-development countries still have room for improvement in bladder-cancer burden. Although the future burden may decrease, the burden in the elderly remains significant. Health inequities across regions also need further addressing. While our study comprehensively analyzed the disease burden of bladder cancer in the elderly, there are still some limitations. First, the analysis relies on the GBD database, whose accuracy is affected by the availability and quality of national registration data. In low- and middle-income countries, insufficient medical resources may lead to incomplete data, with potential misdiagnosis, underdiagnosis, and literature loss. Second, there is a delay in GBD data updates, which prevents a full reflection of the current situation. Additionally, the study mainly analyses the epidemiology of bladder cancer and lacks a comprehensive and in-depth analysis of risk factors, limiting causal inferences. Finally, the GBD data focuses on the overall disease situation and lacks detailed disease information, so we cannot further discuss specific disease subtypes, such as non-muscle-invasive bladder cancer and muscle-invasive bladder cancer.

## Conclusion

In summary, from 1990 to 2021, the bladder cancer burden in people aged 55 and older showed a declining trend. However, significant disparities remain across countries and regions due to unequal development levels. In low SDI regions, the incidence rate for this age group is still increasing, and the decline in mortality and DALYs rates is less pronounced compared to high SDI regions. Future interventions require stratified approaches: implementing stage-tailored early detection in low-SDI regions to address mortality from diagnostic delays, while combating persistently elevated incidence in high-SDI settings through precision metabolic risk management.

## Data Availability

The datasets generated and/or analyzed during the current study are available in the GBD repository and NHANES website (https://www.cdc.gov/nchs/index.htm). For access to the complete dataset and methodological details underpinning this study’s findings, interested researchers may contact the corresponding authors.
